# Increased TCR signal strength in DN thymocytes promotes development of gut TCRαβ^(+)^CD8αα^(+)^ intraepithelial lymphocytes

**DOI:** 10.1038/s41598-017-09368-x

**Published:** 2017-09-06

**Authors:** Capucine L. Grandjean, Nital Sumaria, Stefania Martin, Daniel J. Pennington

**Affiliations:** Blizard Institute, Barts and The London School of Medicine, Queen Mary University of London, London, E1 2AT UK

## Abstract

CD4^(+)^CD8^(+)^ “double positive” (DP) thymocytes differentiate into diverse αβ T cell sub-types using mechanistically distinct programs. For example, conventional αβ T cells develop from DP cells after partial-agonist T cell receptor (TCR) interactions with self-peptide/MHC, whereas unconventional αβ T cells, such as TCRαβ^(+)^CD8αα^(+)^ intraepithelial lymphocytes (IELs), require full-agonist TCR interactions. Despite this, DP cells appear homogeneous, and it remains unclear how distinct TCR signalling instructs distinct developmental outcomes. Moreover, whether TCR signals at earlier stages of development, for example in CD4^(−)^CD8^(−)^ double negative (DN) cells, impact on later fate decisions is presently unknown. Here, we assess four strains of mice that display altered TCR signal strength in DN cells, which correlates with altered generation of unconventional TCRαβ^(+)^CD8αα^(+)^ IELs. FVB/n mice (compared to C57BL/6 animals) and mice with altered preTCRα (pTα) expression, both displayed weaker TCR signalling in DN cells, an inefficient DN-to-DP transition, and reduced contribution of TCRαβ^(+)^CD8αα^(+)^ IELs to gut epithelium. Conversely, TCRαβ^(+)^CD8αα^(+)^ IEL development was favoured in mice with increased TCR signal strength in DN cells. Collectively, these data suggest TCR signal strength in DN cells directly impacts on subsequent DP cell differentiation, fundamentally altering the potential of thymocyte progenitors to adopt conventional versus unconventional T cell fates.

## Introduction

T cell receptor-expressing intraepithelial lymphocytes (IELs) are epithelial-resident T cells found at numerous body locations^1^. Gut IELs display anti-microbial and anti-inflammatory properties and are central to the control of intestinal epithelial homeostasis^2^. A large proportion of gut IELs express TCRαβ, and can be further characterized by expression of CD8αβ or CD8αα dimers^3^. TCRαβ^(+)^CD8αβ^(+)^ IELs are termed “conventional”, closely sharing gene expression signatures with CD8αβ^(+)^ T cells from secondary lymphoid organs^4, 5^. By contrast, “unconventional” TCRαβ^(+)^CD8αα^(+)^ IELs do not require priming in lymphoid structures, appear restricted to the gut epithelium, and display gene expression signatures more similar to γδ T cells^4, 5^.

Initial studies suggested that unconventional TCRαβ^(+)^CD8αα^(+)^ IELs develop extra-thymically in gut lymphoid structures known as cryptopatches^6^. However, TCRαβ^(+)^CD8αα^(+)^ IELs are severely reduced in athymic *nude* mice, and fate-mapping experiments suggested they traverse the CD4^(+)^CD8^(+)^ double positive (DP) stage in the thymus^7^. Further work implicated agonist self-peptide-mediated selection through TCRαβ at the DP stage^8^, and identified pre- and post-selection progenitor subsets^9^. Nonetheless, it remains unclear how “strong” TCR-agonist signals in DP cells instruct positive selection of TCRαβ^(+)^CD8αα^(+)^ IELs instead of driving negative selection. Indeed, unconventional TCRαβ^(+)^CD8αα^(+)^ IELs were recently found to express TCRs that had been “recycled” from strong negatively selecting signals^10^.

Although TCRαβ signalling at the DP stage appears critical for TCRαβ^(+)^CD8αα^(+)^ IEL development, the DP stage is not the first in which TCR signalling occurs. DP cells arise from CD4^(−)^CD8^(−)^ double negative (DN) cells in a process known as “β-selection” that is mediated by signalling through the preTCR (rearranged TCRβ paired with invariant pTα)^11^. PreTCR signalling is generally considered weak, due to very low surface preTCR expression^12^. By contrast, stronger signalling in DN cells, for example by TCRγδ, is less efficient at generating DP cells; instead driving cells to a γδ T cell fate^13, 14^.

Successful transition through the β-selection checkpoint results in cell survival, extensive proliferation, and significant differentiation, events that may be mechanistically linked^15^. Although TCR signal strength in DN cells clearly affects the efficient induction of these processes, it is presently unclear whether it also affects the future fate of the DP cells that are generated. Here, we begin to investigate this idea in the context of TCRαβ^(+)^CD8αα^(+)^ IEL development. We show that FVB/n wild type (WT) mice have a much reduced TCRαβ^(+)^CD8αα^(+)^ IEL compartment when compared with WT C57BL/6 animals, that correlates with weaker preTCR signalling at the β-selection checkpoint. Indeed, by reducing preTCR signal strength in pTα-transgenic animals we re-capitulate this relative absence of gut TCRαβ^(+)^CD8αα^(+)^ IELs. By contrast, in two mouse models in which TCR signal strength is greater in DN cells by forced expression of TCRαβ, increased generation of TCRαβ^(+)^CD8αα^(+)^ IELs was observed. Thus, these data provide evidence that TCR signal strength at the DN-to-DP transition directly influences the efficiency of TCRαβ^(+)^CD8αα^(+)^ IEL development.

## Results

### Reduced TCRαβ^(+)^CD8αα^(+)^ IELs in FVB/n mice

**Figure 1:**
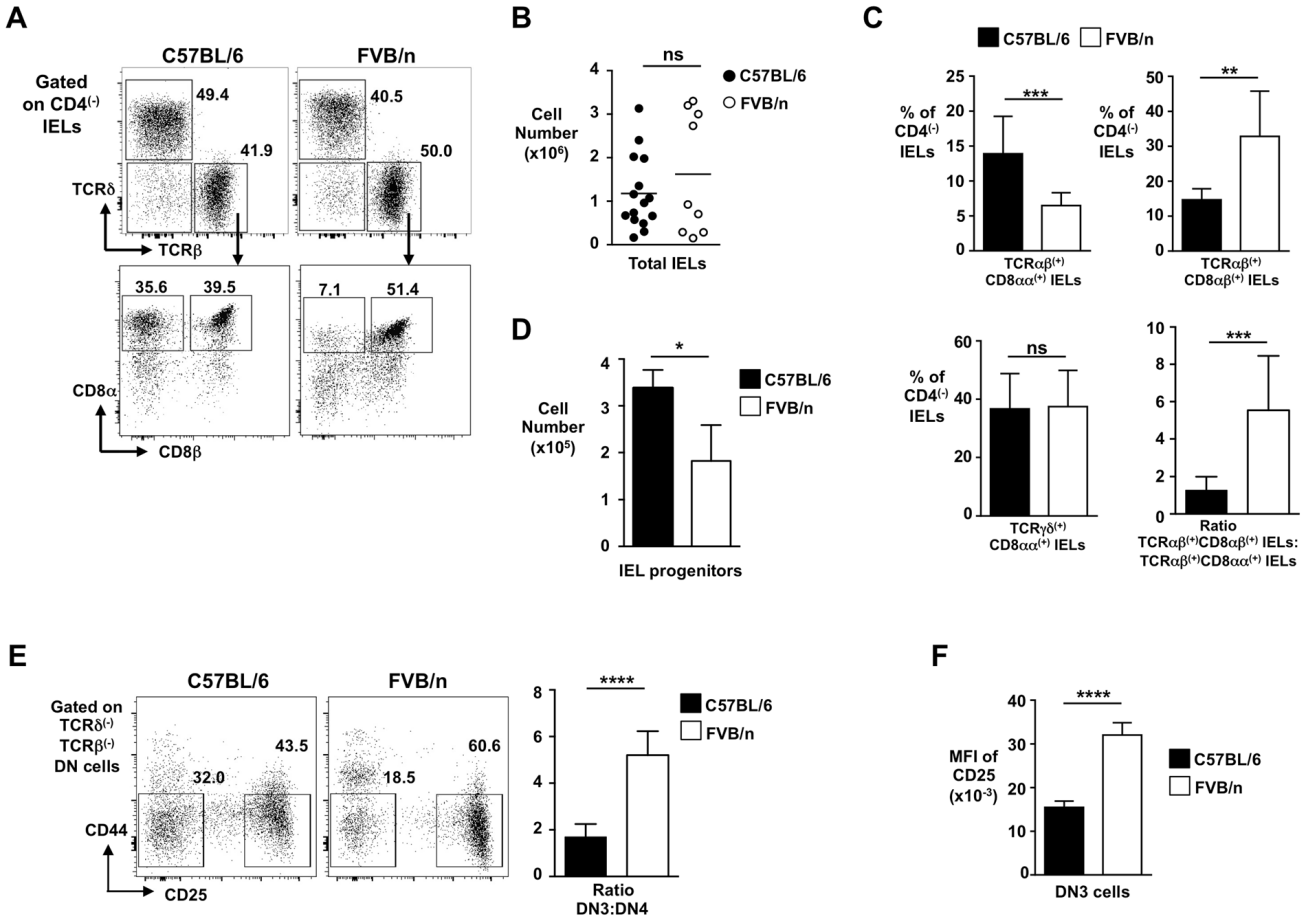
Reduced TCRαβ^(+)^CD8αα^(+)^ IELs in FVB/n mice. (**A**) Representative flow cytometry plots (from n > 6) of IEL populations (TCRαβ^(+)^CD8αα^(+)^ and TCRαβ^(+)^CD8αβ^(+)^ IELs) from small intestine of C57BL/6 and FVB/n mice. Gating strategy and percentages of cells are indicated. (**B**) Total cell yield for IEL-preps from C57BL/6 and FVB/n mice. (**C**) Summary bar graphs (n > 6) of percentages (of total CD4^(−)^ IELs) of TCRαβ^(+)^CD8αα^(+)^, TCRαβ^(+)^CD8αβ^(+)^ and TCRγδ^(+)^CD8αα^(+)^ IELs from C57BL/6 and FVB/n mice. Right-hand bottom graph shows ratio of conventional TCRαβ^(+)^CD8αβ^(+)^ to unconventional TCRαβ^(+)^CD8αα^(+)^ IELs. (**D**) Bar graph showing total cell number of thymic IEL progenitors from C57BL/6 and FVB/n mice gated as CD4^(lo)^CD8^(lo)^TCRδ^(−)^TCRβ^(+)^CD5^(hi)^PD-1^(+)^CD69^(+)^CD122^(+)^ cells (n > 5). (**E**) Representative flow cytometry plots (from n > 10) of thymic DN3 and DN4 subsets (cells shown gated as CD4^(−)^CD8^(−)^TCRβ^(−)^TCRδ^(−)^) from C57BL/6 and FVB/n mice, with summary bar chart for the DN3 to DN4 ratio in both strains. (**F**) Summary bar chart of mean fluorescence intensity (MFI) of surface CD25 on DN3 thymocytes from each strain of mice.

We had noted that FVB/n wild type (WT) mice, in comparison with C57BL/6 WT mice, had significantly reduced unconventional TCRαβ^(+)^CD8αα^(+)^ IELs in the small intestine (Fig. 1A) (total IEL yields from the two strains were variable but not significantly different (Fig. 1B)). By contrast, the proportion of conventional TCRαβ^(+)^CD8αβ^(+)^ IELs was increased in FVB/n mice, and TCRγδ^(+)^ IELs were comparable between the two strains (Fig. 1C). Unconventional TCRαβ^(+)^CD8αα^(+)^ IELs are thought to develop in the thymus from cells that are CD4^(lo)^CD8^(lo)^TCRδ^(−)^TCRβ^(+)^CD5^(hi)^CD69^(+)^PD-1^(+)^CD122^(+)^ (Sup Figure S1)^16^. C57BL/6 mice possess ~3.5 × 10^5^ of these cells. However, consistent with a reduced unconventional IEL compartment, the comparative subset of FVB/n mice was significantly lower at ~2 × 10^5^ cells (Fig. 1D). IEL progenitors develop from CD4^(+)^CD8^(+)^ double positive (DP) cells^9, 16^, that in turn are generated when the preTCR (rearranged TCRβ paired with invariant pre-Tα) drives CD44^(−)^CD25^(+)^ DN3 cells to CD44^(−)^CD25^(−)^ DN4 cells (and then on to the DP stage) in a process called β-selection^11^. C57BL/6 mice had an expected DN3-to-DN4 ratio of ~1.5, consistent with a normal transition through the β-selection checkpoint (Fig. 1E). However, the DN3-to-DN4 ratio of FVB/n was over twice as large, reminiscent of mice lacking a component of the preTCR (e.g. pTα^−/−^ mice^11^). Moreover, DN3 cells from FVB/n mice had significantly elevated surface expression of CD25, a signatory feature of inefficient progression through the DN-to-DP transition^11^ (Fig. 1F). Thus, FVB/n mice, that have a significantly reduced unconventional TCRαβ^(+)^CD8αα^(+)^ IEL compartment compared with C57BL/6 animals, also display inefficient developmental progression through thymic stages that lead to generation of unconventional IEL progenitors.

### Weak preTCR signal strength in FVB/n mice underlies inefficient β-selection

**Figure 2:**
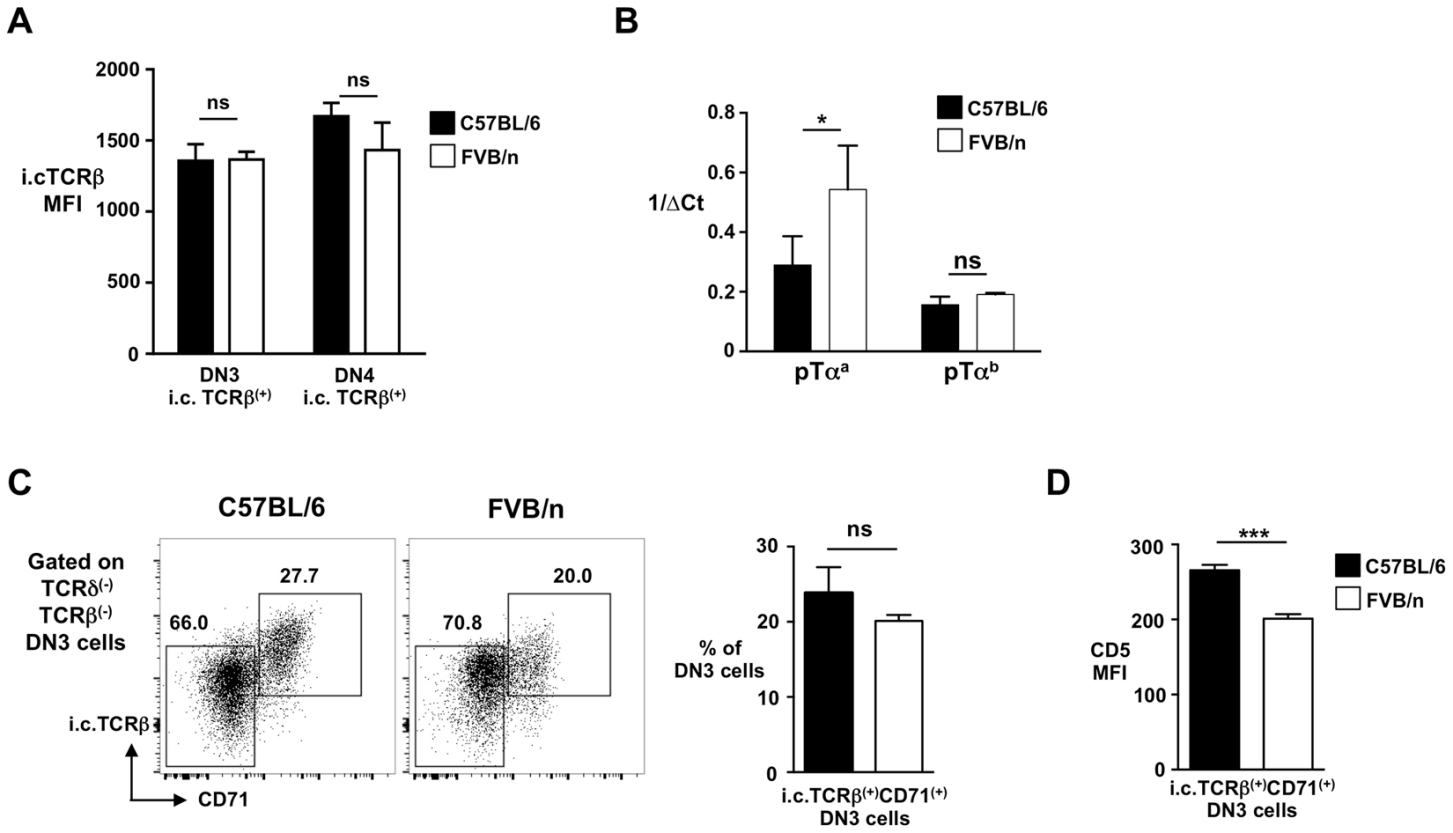
Weak preTCR signal strength in FVB/n mice underlies inefficient β-selection. (**A**) Summary bar graph of i.c.TCRβ MFI in DN3 and DN4 thymocytes from C57BL/6 and FVB/n mice. (**B**) Relative level of pTα^a^ and pTα^b^ transcripts expressed by the DN3 thymocytes from C57BL/6 and FVB/n mice. ΔCt corresponds to normalised CT value of reaction according to the reference gene GAPDH. *p < 0.05, ns is for not significant. (**C**) Representative flow cytometry profiles (left) showing i.c.TCRβ and CD71 on DN3 cells from C57BL/6 and FVB/n mice. Percentages of cells are indicated. Summary bar chart (right) shows percentage of DN3 cells that are i.c.TCRβ CD71^(+)^. (**D**) CD5 MFI levels on CD71^(+)^ β-selected DN3 thymocytes (i.c.TCRβ^(+)^CD71^(+)^) from C57BL/6 and FVB/n mice. ****p < 0.0001, ***p < 0.001, **p < 0.01, ns is for not significant.

To investigate the inefficient DN3-to-DN4 transition in FVB/n mice that appeared to correlate with reduced unconventional TCRαβ^(+)^CD8αα^(+)^ IEL development, we first assessed components of the preTCR. Intracellular TCRβ levels in DN3 cells appeared comparable between FVB/n and C57BL/6 animals (Fig. 2A). For pTα, two isoforms exist; a full-length pTα^a^, and truncated pTα^b^, both of which can form a preTCR and drive β-selection^17^. Interestingly, single cell PCR of both the DN3 and DN4 subsets revealed examples of single cells expressing pTα^a^ alone, pTα^b^ alone, or both pTα^a^ and pTα^b^ (Sup Figure S2). When the strains were compared, DN3 cells from FVB/n mice appeared to express significantly more pTα^a^ (but not more pTα^b^), than C57BL/6 mice (Fig. 2B). It was shown that preTCR consisting of pTα^a^:TCRβ is expressed at significantly lower surface levels than preTCR consisting of pTα^b^:TCRβ, and that this lower surface expression results in decreased preTCR signal strength^17^. Consistent with this, FVB/n mice possessed a lower proportion (approaching significance) of CD71^(+)^i.c.TCRβ^(+)^ proliferating DN3 cells^18^ (Fig. 2C), that expressed significantly lower levels of CD5 (Fig. 2D), a recognised marker of TCR signal strength^19^, than similar cells from C57BL/6 mice, suggesting that inefficient β-selection may be due to weak preTCR signalling in FVB/n animals. Thus, weaker signalling during the DN-to-DP transition correlates with reduced numbers of unconventional TCRαβ^(+)^CD8αα^(+)^ IELs in FVB/n mice.

### Reduced unconventional TCRαβ^(+)^CD8αα^(+)^ IELs in pTα^a^-only transgenic mice

**Figure 3:**
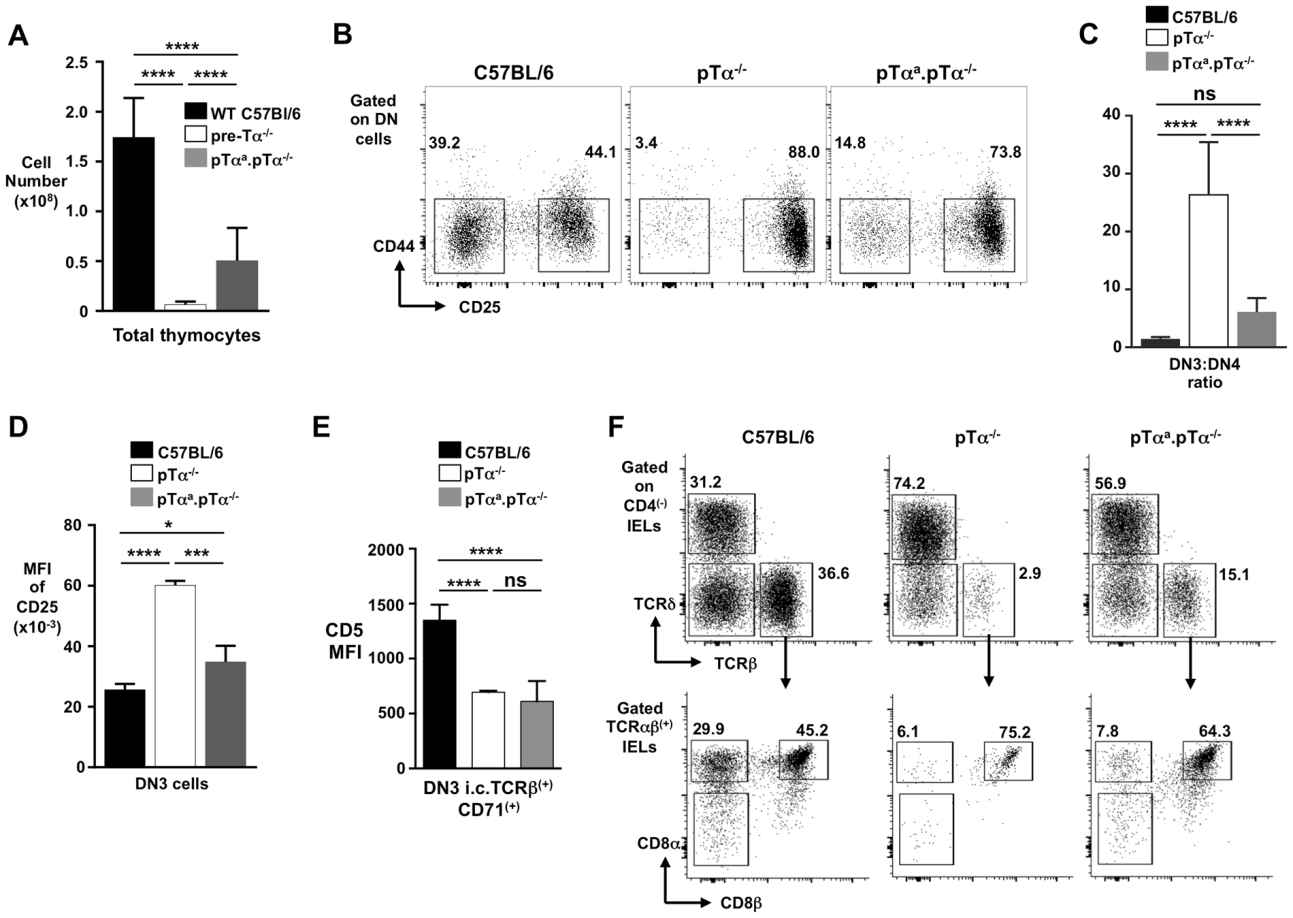
Reduced unconventional TCRαβ^(+)^CD8αα^(+)^ IELs in pTα^a^-only transgenic mice. (**A**) Summary bar chart of DP cell numbers in C57BL/6, pTα-deficient and pTα^a^. pTα^−/−^mice. (**B**) Representative flow cytometry profiles of DN3 and DN4 subsets from thymus of C57BL/6, pTα^−/−^ and pTα^a^. pTα^−/−^ transgenic mice. (**C**) Bar chart summarizing the DN3 to DN4 ratio in C57BL/6, pTα-deficient and pTα^a^. pTα^−/−^mice. (**D**) Summary bar chart of MFI of CD25 on DN3 thymocytes from each strain of mice. (**E**) Bar chart showing CD5 MFI on β-selected DN3 cells (i.c.TCRβ^(+)^CD71^(+)^) from each strain of mice. (**F**) Representative flow cytometry plots (from n > 6) of IEL populations (TCRαβ^(+)^CD8αα^(+)^ and TCRαβ^(+)^CD8αβ^(+)^ IELs) from the small intestine of C57BL/6, pTα-deficient and pTα^a^. pTα^−/−^ mice. Percentages of cells are indicated near each gate. ****p < 0.0001, ***p < 0.001, *p < 0.05, ns is for not significant.

To test the idea that weaker signalling at the β-selection checkpoint and reduced generation of unconventional TCRαβ^(+)^CD8αα^(+)^ IELs are linked, we generated mice in which only a weakly signalling pTα^a^-containing preTCR could be formed. pTα-intron-1 was removed from the full pTα genomic locus (to prevent splicing to pTα^b^), in a 50 kb bacterial artificial chromosome (BAC), that was then used to generate pTα^a^-only BAC transgenic mice on a pTα^−/−^ (C57BL/6) background (Sup Figure S3A). As expected, the pTα^a^-only transgene largely rescued the pTα^−/−^associated block in thymocyte development as assessed by comparison of thymus and spleen profiles from C57BL/6 WT, pTα^−/−^, and pTα^a^. pTα^−/−^ mice (Sup Figure S3B). However, total thymocyte number in pTα^a^. pTα^−/−^ mice, as reflected by the DP subset, did not reach WT levels, (Fig. 3A). Consistent with this, transition through the β-selection checkpoint was markedly reduced (Fig. 3B), with a DN3:DN4 ratio of ~5 in pTα^a^. pTα^−/−^ mice compared to ~1 in WT animals (Fig. 3C). Moreover, surface CD25 was elevated on DN3 cells from pTα^a^. pTα^−/−^ animals, compared to WT (Fig. 3D), while newly selected proliferating CD71^(+)^ DN3 cells displayed markedly less CD5, indicative of weaker TCR signalling (Fig. 3E). Importantly, this evidence of an inefficient DN3-to-DN4 transition in pTα^a^. pTα^−/−^ mice correlated with a notable absence of unconventional TCRαβ^(+)^CD8αα^(+)^ IELs in the small intestine (Fig. 3F) (total IEL cell yield was not significantly different between the three strains (Sup Figure S4A)). By contrast, conventional TCRαβ^(+)^CD8αβ^(+)^ IELs and TCRγδ^(+)^ IELs were present in normal proportions (Sup Figure S4B). Thus, pTα^a^. pTα^−/−^ mice, that can only generate a pTα^a^-containing preTCR that signals weakly, display inefficient transition through the β-selection checkpoint, and a significant absence of unconventional TCRαβ^(+)^CD8αα^(+)^ IELs.

### Strong TCR signalling in DN cells promotes increased development of unconventional TCRαβ^(+)^CD8αα^(+)^ IELs

**Figure 4:**
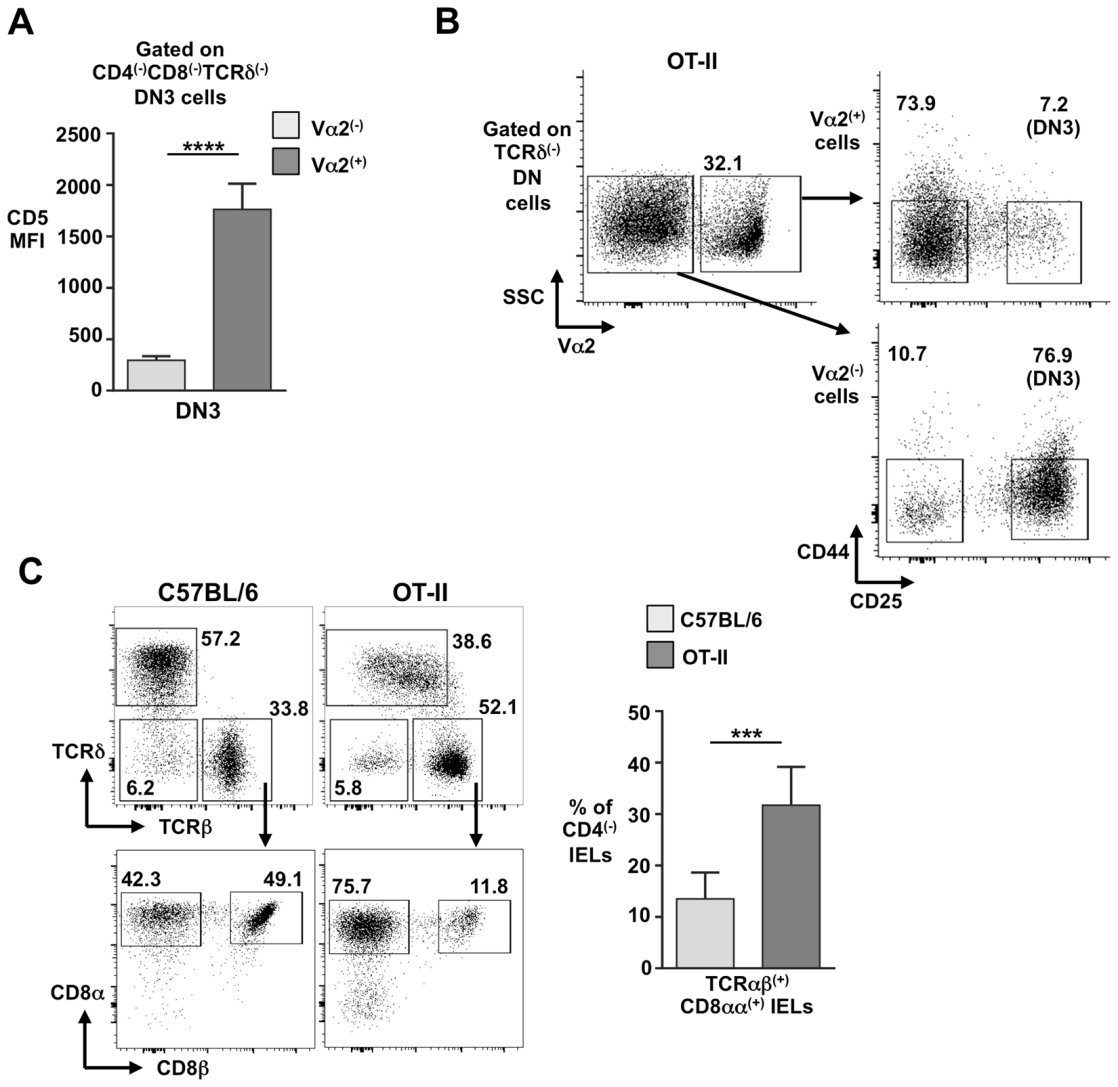
Strong TCR signalling in DN cells of OT-II TCR transgenic mice promotes increased development of unconventional TCRαβ^(+)^CD8αα^(+)^ IELs. (**A**) Summary bar chart of CD5 MFI on Vα2^(−)^ and Vα2^(+)^ DN3 thymocytes from TCR-transgenic OT-II mice. (**B**) Representative flow cytometry profiles of Vα2^(−)^ and Vα2^(+)^ DN3 and DN4 subsets from the thymus of TCR-transgenic OT-II mice. (**C**) Representative flow cytometry plots for TCRαβ^(+)^ IEL populations (TCRαβ^(+)^CD8αα^(+)^ and TCRαβ^(+)^CD8αβ^(+)^ IELs) from the small intestine of C57BL/6 and OT-II mice. Right is summary bar chart. Percentages of gated cells are indicated. ****p < 0.0001.

The above data suggest that weak TCR signalling at the β-selection checkpoint leads to a subsequently less efficient development of unconventional TCRαβ^(+)^CD8αα^(+)^ IELs. To test this idea further, we sought to increase TCR signalling in DN cells to ascertain if this would conversely increase unconventional TCRαβ^(+)^CD8αα^(+)^ IEL development. TCRαβ transgenic (tg) mice aberrantly express TCRαβ complexes that signal strongly at the β-selection checkpoint. Indeed, as well as driving DN cells to the DP stage, this strong TCR signalling is also thought to divert some DN progenitors to the γδ lineage^20–22^. OT-II mice (C57BL/6 background), express an ovalbumin-specific MHC-II-restricted Vα2^(+)^Vβ5^(+)^ transgenic TCRαβ^23^. Vα2^(+)^ (i.e. largely tg-TCR^(+)^) DN3 cells from OT-II mice express much higher levels of CD5 than Vα2^(−)^ DN3 cells, indicative of stronger TCR signalling (Fig. 4A). And in contrast to DN cells from FVB/n and pTα^a^. pTα^−/−^ mice, Vα2^(+)^ DN cells from OT-II mice traverse the β-selection checkpoint efficiently (Fig. 4B). Importantly, on inspection of the gut of OT-II mice, a significantly increased proportion of unconventional TCRαβ^(+)^CD8αα^(+)^ IELs was observed, notably inverting the TCRαβ^(+)^CD8αα^(+)^ IEL-to-TCRαβ^(+)^CD8αβ^(+)^ IEL ratio (Fig. 4C).

**Figure 5:**
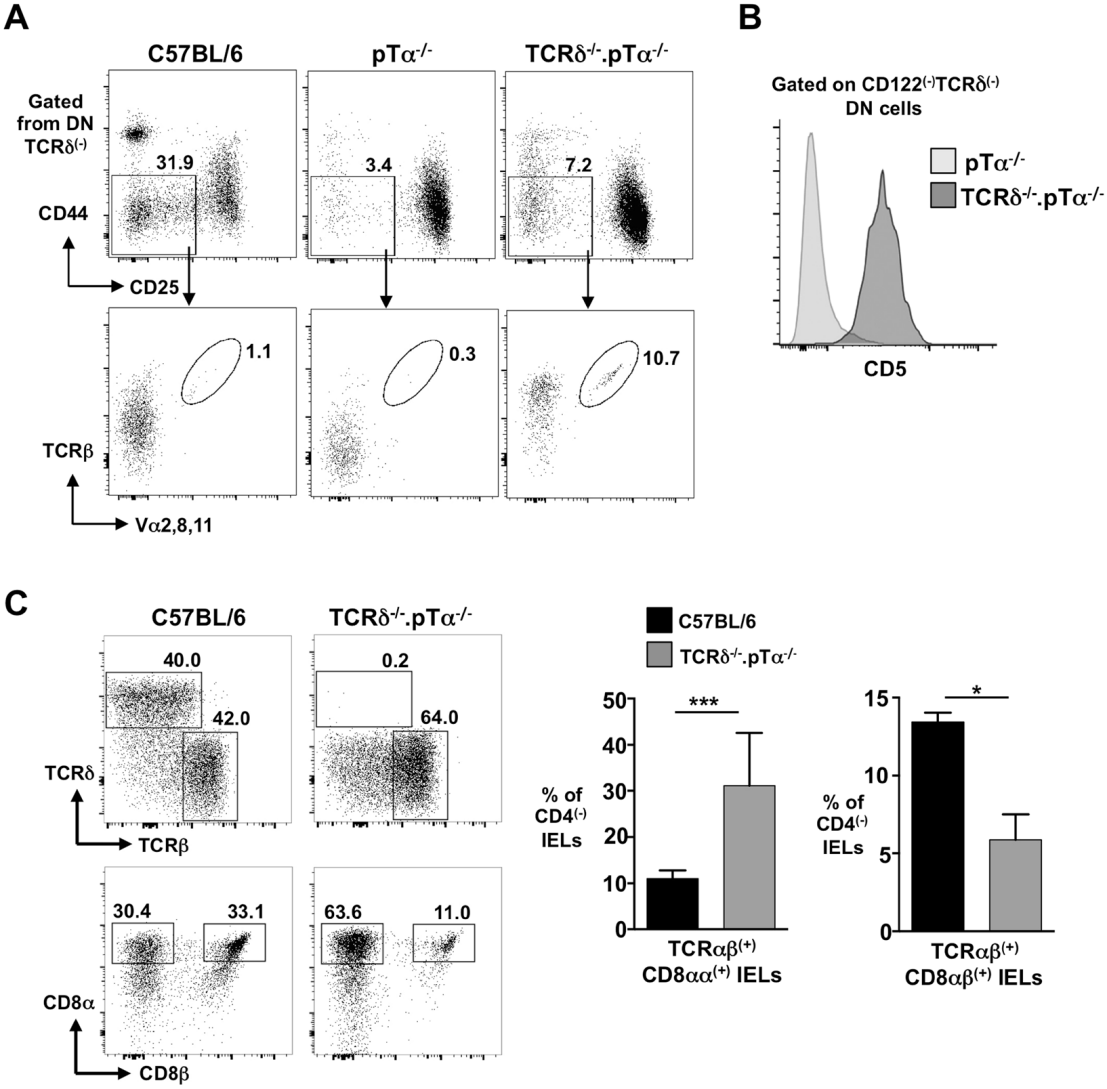
Strong TCR signalling in DN cells of TCRδ^−/−^. pTα^−/−^ mice promotes increased development of unconventional TCRαβ^(+)^CD8αα^(+)^ IELs. (**A**) Representative flow cytometry plots of DN3 and DN4 subsets from C57BL/6, pTα^−/−^ and TCRδ^−/−^. pTα^−/−^ mice. Lower plots show surface TCRβ, and combined expression of Vα2, Vα8 and Vα11, on DN4 cells from each strain. (**B**) Representative (from n > 3) histogram of CD5 on CD122^(−)^TCRδ^(−)^ DN cells from pTα^−/−^ and TCRδ^−/−^. pTα^−/−^ mice. (**C**) Representative flow cytometry plots and corresponding summary bar charts of TCRαβ^(+)^ IEL populations from C57BL/6, and pTα^−/−^. TCRδ^−/−^ mice. Percentages of gated cells are indicated. ****p < 0.0001.

TCR-tg mice express a single TCRα/TCRβ combination that may have unusual signalling characteristics. Thus, to extend these studies, we took advantage of a little-known feature of TCRδ^−/−^ mice; the rearrangement and expression of early TCRα transcripts induced by the strong PGK promoter used to delete the Cδ region (Sup Figure S5)^24, 25^. We crossed TCRδ^−/−^ mice with pTα^−/−^ mice to generate TCRδ^−/−^. pTα^−/−^ mice in which DN cells cannot express either TCRγδ or preTCR, yet can (in some cells) rearrange early TCRα (and TCRβ). Compared with pTα^−/−^ mice, TCRδ^−/−^. pTα^−/−^ mice had an increased population of DN4 cells, the majority of which robustly expressed TCRβ (Fig. 5A). Unlike for TCRβ, a pan-TCRα antibody is not available. However, when antibodies for Vα2, Vα8 and Vα11 were used together over 10% of DN4 cells from TCRδ^−/−^. pTα^−/−^ mice stained positive compared with <0.5% of DN4 cells from pTα^−/−^ animals (Fig. 5A). Moreover, DN cells from TCRδ^−/−^. pTα^−/−^ mice expressed high levels of CD5 consistent with stronger TCRαβ-driven signalling (Fig. 5B). Thus, β-selection in TCRδ^−/−^. pTα^−/−^ mice is driven solely by a broad repertoire of early-expressed TCRαβ complexes. Importantly, on inspection of the small intestine of TCRδ^−/−^. pTα^−/−^ mice, an increased proportion of unconventional TCRαβ^(+)^CD8αα^(+)^ IELs was observed, again inverting the TCRαβ^(+)^CD8αα^(+)^ IEL-to-TCRαβ^(+)^CD8αβ^(+)^ IEL ratio (Fig. 5C). Collectively, these data support the hypothesis that TCR signal strength in DN cells at the β-selection checkpoint influences subsequent unconventional TCRαβ^(+)^CD8αα^(+)^ IEL development.

## Discussion

The developmental origins of TCRαβ^(+)^CD8αα^(+)^ intraepithelial lymphocytes have long been debated. Initially, the recognition that TCRαβ^(+)^CD8αα^(+)^ IELs, but not TCRαβ^(+)^CD8αβ^(+)^ IELs, were present in athymic mice led to suggestion of their extra-thymic generation in gut lymphoid structures called cryptopatches (CPs)^6^. Indeed, CPs were shown to contain CD25^(+)^IL-7Rα^(+)^c-kit^(+)^ progenitors that could reconstitute the T cell compartments of irradiated T cell-deficient animals^26^. Nonetheless, that athymic mice have only 5–10% of the total TCRαβ^(+)^CD8αα^(+)^ IELs of euthymic mice conversely argued for a predominately thymic origin. The thymic generation of TCRαβ^(+)^CD8αα^(+)^ IELs was further supported by fate mapping experiments in which a GFP marker was activated by RORγt-promoter-driven Cre protein^7^. RORγt is expressed in thymic DP cells but not in their DN precursors. As all γδ T cells develop from DN cells, TCRγδ^(+)^ IELs were GFP^(−)^. By contrast, TCRαβ^(+)^CD8αα^(+)^ IELs were GFP^(+)^, suggesting their development through a DP stage^7^.

It was also recognized early that TCRαβ^(+)^CD8αα^(+)^ IELs use “forbidden” TCRβ chains that were purged from the conventional TCRαβ^(+)^ T cell pool by superantigen-driven negative selection of DP cells in the thymus^27^. Nonetheless, despite evidence of “TCR-agonist-selection”^8, 28^, identifying the thymic stages through which TCRαβ^(+)^CD8αα^(+)^ IEL progenitors progress has proven more problematic. Cheroutre and colleagues described a minor subset of CD8αα^(+)^ DP cells that appeared to develop as TCRαβ^(+)^CD8αα^(+)^ IELs if exposed to TCR-agonist signals^9^. Somewhat surprisingly, these “triple positive” (TP) cells then shut off CD4, CD8α and CD8β to become TCRαβ^(+)^CD5^(+)^ DN cells, before re-expressing CD8αα^(+)^ in the gut epithelium possibly under the influence of TGFβ^29^. By contrast, the stages that precede the TP stage are less clear. As too are the reasons why TP cells survive TCR-agonist signals, rather than dying as DP cells do during TCR-agonist-driven negative selection. It is conceivable that TP cells have already entered an unconventional IEL developmental pathway, and are thus cell-intrinsically distinct from DP cells that allow them to survive agonist signals. In this regard, RhoH^30^, and TGFβ signalling^29^, have been implicated in differential thymic development of TCRαβ^(+)^CD8αα^(+)^ IELs versus conventional T cell subsets. Nonetheless, how unconventional IEL progenitors could adopt such a distinct gene expression profile by the TP stage, and indeed how TP cells are even generated, had not been previously elucidated.

In this study, we have shown that modulation of TCR signal strength at the thymic DN stage fundamentally impacts on the development of TCRαβ^(+)^CD8αα^(+)^ IELs; stronger TCR signalling in DN cells, as judged by CD5 surface levels and the efficiency of the DN-to-DP transition, appearing to favour TCRαβ^(+)^CD8αα^(+)^ IEL generation. As TP cells, like DP cells, are presumably derived from DN precursors as a result of (pre)TCR signalling, we speculate that stronger signalling at the DN stage favours TP generation. Unfortunately, we were unable to formally test this idea, as CD8αα-specific antibodies are not available and TL-tetramer staining did not work in our hands^9^. Nonetheless in this regard, peripheral CD4^(+)^ cells do upregulate CD8αα when activated through TCRαβ (by cross-linking antibodies) in the presence of TGFβ^29^, the absence of which has been correlated with reduced generation of TCRαβ^(+)^CD5^(+)^ IEL precursors^29^.

At first glance, invoking strong TCR signals in DN cells to drive generation of (CD8αα^(+)^) DP cells conflicts with the notion that such signals are required for commitment to the γδ T cell lineage^13, 14^. Indeed, premature expression of TCRα in DN cells of TCRαβ transgenic mice was shown to promote differentiation of CD4^(−)^CD8^(−)^TCRαβ^(+)^ cells with γδ T cell-like properties^20–22^. Nonetheless, many of these studies also reported increased numbers of TCRαβ^(+)^CD8αα^(+)^ IELs, and made obvious but perhaps incorrect links between these DN TCRαβ^(+)^ cells and IEL development^31^. However, these “γδ-wannabie” cells do not traverse the DP stage (unlike *bone fide* IEL progenitors), and do not rearrange their endogenous TCRα chains^20^; something obviously required for TCRαβ^(+)^ IEL development. By way of explanation, our data now suggest that in TCRαβ^(+)^ transgenic mice at least some DN cells will transition to the DP stage as a result of stronger transgenic-TCR signalling. This could boost the TP cell to conventional DP cell ratio, which in turn would favour increased TCRαβ^(+)^CD8αα^(+)^ IEL development.

Finally, what would normally provide the stronger TCR signals in DN cells to drive TP generation in the absence of prematurely expressed transgenic TCRαβ? TCRα is reported to rearrange in a small fraction of WT DN cells^32^, although whether this is physiologically relevant is unclear. Instead, a candidate “strong-signalling” TCR could be preTCR^b^ that is generated when rearranged TCRβ binds to the truncated pTα^b^ isoform that lacks the pTα extracellular Ig-domain encoded by exon-2 ^17^. PreTCR^b^ was shown to signal more strongly than preTCR^a^ due to the higher preTCR^b^ surface expression levels permitted by pTα^b^ ^17^. Such stronger signalling may result in a higher proportion of DN cells becoming TP cells. Our data presented here are consistent with such a mechanism. BAC-tg mice in a pTα^−/−^ background, that expressed only pTα^a^ under the physiological pTα promoter, showed weaker than normal TCR signal strength in DN cells, a relatively inefficient DN-to-DP transition, and a reduced proportion of unconventional TCRαβ^(+)^CD8αα^(+)^ IELs.

In sum, our findings shed new light on the early thymic stages of unconventional IEL development. We demonstrate that TCR signal strength in DN cells at the β-selection checkpoint significantly influences the development of unconventional TCRαβ^(+)^CD8αα^(+)^ IELs. Stronger signalling favours TCRαβ^(+)^CD8αα^(+)^ IEL development. By contrast, weaker signalling favours a greater contribution from conventional TCRαβ^(+)^CD8αβ^(+)^ IELs that are likely differentiated from naïve conventional CD8αβ^(+)^ T cells in the gut-associated lymphoid tissue.

## Methods

All experimental protocols were performed in, and approved by, The Blizard Institute, Bart’s and The London School of Medicine, Queen Mary University of London.

### Mice

C57BL/6 and FVB/n mice were purchased from Charles River Laboratories. All mice were 6–12 weeks old. Mice were bred and maintained in specific pathogen-free animal facilities at Queen Mary University of London. All experiments were performed in compliance with relevant laws and institutional guidelines and were approved by a local ethics committee.

### Generation of pTα^a^. pTα^−/−^ mice

BAC-transgenic mice were generated by the recombineering technique (http://recombineering.ncifcrf.gov)^33^. In brief, a pTα locus-containing BAC (BMQ452P20) was purchased from www.ensemble.org. A GalK cassette was first amplified from a GalK plasmid using primers that contained 50 bp homology with sections of pTα intron-1;

Fwd:5′GACAGGGTTTCTCTGTGTAGCTCCGGCTGTCCT GGAACTCACTCTGAGACCAGGCTGGCCTCGAACTCAGA AATCCTGTTGACAATTAATCATCGGCA-3′;

Rev: 5′TGGGTTGTTGTGGGTGGGCGGTTGTTAGTTGGTTG CTGTCAGTCTTGGCTTGCTAA GTAGTCGTGGGCAAAGAATCAGCACTGTCCTGCTCCTT-3′.

The GalK PCR product was then inserted by homologous recombination into intron-1 and selected for in minimal broth containing galactose. Removal of intron-1 was mediated by a double-stranded oligo made by annealing two primers with 75 bp homology to the targeted regions;

Fwd:5′TGGGTCATGCTTCTCCACGAGTGGGCCATGGCTAGGA CATGGCTGCTGCTGC TTCTGGGCGTCAGGTGTCAGGCCCTACCATCA GGCATCGCTGGCACCC-3′;

Rev:5′AGGCAAACCACCAGCATGTGCTGCCTTCCATCTACCAGC AGTGTGATGGG TGGAGCCAGAGACGGAAAGGGGGTG CCAGCGATGCCTGATGGTAGGGCCT-3′.

The resulting BAC was selected for removal of the GalK gene. After sequencing, a 28 kb Spe1 fragment was used for injection into the pro-nuclei of fertilized C57BL/6 oocytes.

### Tissue processing and cell isolation

For single-cell suspensions, thymus and spleen were crushed and filtered through 80μm stainless steel mesh (Sefar Ltd., UK) in fluorescence-activated cell sorting (FACS) buffer (phosphate-buffered saline (PBS) containing; 2% heat-inactivated fetal calf serum (FCS) (Life technologies); and 5 mM ethylenediaminetetraacetic acid (EDTA) (Life technologies)). For spleen, red blood cells were removed by gradient centrifiguation at 1600rpm for 25 min using 4 mL of Lymphocyte Separation Medium (Fischer). For IEL preparations, faecal material was flushed from lumen of small intestine with ice-cold PBS using a gavage needle. Fatty tissues, vasculature and Peyer’s patches were removed, followed by longitudinal opening and 60 min agitation in RPMI-1640 10% Newborn calf serum (NCS) (Life technologies) with 5 mM EDTA (Life technologies) at 37 °C. Cells were subsequently passed through an autoclaved column containing 0.7 g of nylon wool (Polysciences, USA), equilibrated with RPMI-1640 with 38 mM HEPES (Life technologies). IELs were enriched on a discontinuous Percoll gradient (40%/80% isotonic Percoll), before use and stained as described below.

### Flow cytometry and cell sorting

Fluorochrome-conjugated antibodies (eBioscience or BD) were; CD3ε (145-2C11), CD4 (RM4-5), CD8α (53–6.7), CD25 (PC61), CD44 (IM7), TCRδ (GL3), TCRβ (H57–597), CD8β (H35–17.2), CD5 (53–7.3), CD69 (H1.2F3), Vα2 (B20.1), CD117 (2B8), B220 (RA3–6B2). For surface staining, cells were Fc-blocked (2.4G2; eBioscience) and stained with antibodies in FACS buffer (PBS with 2% FCS, Life Technologies) for 30 mins. Cells were then washed twice with FACS buffer using a centrifugation speed of 300 g for 5 mins. After staining, cells were resuspended in FACS buffer containing 0.5 μg/ml DAPI (Life technologies) for dead cell exclusion. For intracellular staining, cells were first stained for extracellular markers and subsequently fixed and permeabilized using the eBioscience intracellular flow kit according to the manufacturer’s protocol. The corresponding isotype control was used as a negative control. Samples were acquired using an LSR-II and analysed using Flow Jo v10.

### RNA isolation, cDNA production and real-time PCR

Total RNA was extracted using the RNeasy Mini kit (QIAGEN) according to the manufacturer’s instructions. Concentration and purity was determined using the NanoDrop ND-1000 spectrophotometer (Thermo Scientific). Total RNA was reverse-transcribed into cDNA using the High-Capacity RNA-to-cDNA™ Kit (life technologies). Quantitative real-time PCR was performed using the power SYBR green PCR Master mix (life technologies) on the 7500 real-time PCR system (life technologies). Primers were designed manually and recognised intron/exon boundaries;

pTα^a^Fwd: 5′-GGCTCTACCATCAGGCATCGC-3′;

pTα^a^Rev: 5′-GGTGGTTTGCCTGGTCCTCG-3′;

pTα^b^Fwd: 5′-GCTCTACCATCAGGGGGAATCTTC-3′;

pTα^b^Rev: 5′-CGGGGGGACACAGCGG-3′.

All samples were run in triplicate and expression was normalised against the housekeeping gene GAPDH. Analysis of PCR results was performed on 7500 real-time software v2.0.6 (life technologies).

### Single cell PCR

Cells were individually FAC-sorted into 96 well-plates containing 10 μl of reverse transcription mix (2% Triton-X, 10U RNAseOUT (Life Technologies), 30U MMLV-RT reverse transcriptase (life Technologies), 1% BSA and 0.5 μM of the following primers;

pTαgeneF: 5′-TAGGACATGGCTGCTGCTGC-3′;

pTαgeneR: 5′-TCCCACCCACAGAATTTGGAC-3′.

Plates were incubated for 2hr at 37 °C in a thermocycler (Biorad) and the reaction was stopped by 10 min incubation at 70 °C. Two rounds of PCR were then carried out on the cDNA. The first round of PCR consisted of denaturation at 94 °C, followed by 34 cycles of amplification (20 sec at 94 °C, 45 sec at 52 °C and 1 min at 72 °C) in a PCR mix containing 7.5U of Taq polymerase (NEB), 0.25 mM dNTPs (NEB), 1.6 mM MgCl_2_ (NEB) and 0.25 mM of the pTαgeneF and pTαgeneR primers. The second round PCR was performed using 2.0 μl of the PCR product generated by the first-round PCR and consisted of a 3 min denaturation step followed by 25 cycles of amplification (20 sec at 94 °C, 45 sec at 52 °C and 1 min at 72 °C) in a PCR mix containing 6.5U of Taq polymerase, 1.6 mM MgCl_2_, 0.25 mM dNTPs, and 0.25 mM of the following primers;

pTα^a^F: 5′-GCTCTACCATCAGGCATCGC-3′;

pTα^a^R: 5′-CTCCAGCTGTCAGGGGAATC-3′;

pTα^b^F: 5′-GCTCTACCATCAGGGGAATCTTC-3′;

pTα^b^R: 5′-GCAGGTACTGTGGCTGAGC-3′.

PCR products were run on a ReadyAgarose 96 Plus gel (Biorad) and PCR products were sequenced for validation.

### Statistical analysis

Data are presented as mean ± s.d. A student’s t-test was used to assess statistical significance between groups. A difference was considered significant if p < 0.05.

## Electronic supplementary material

Supplementary Figures
