# Supplementary material for: Increased TCR signal strength in DN thymocytes promotes development of gut TCRαβ(+)CD8αα(+) intraepithelial lymphocytes
Source: Sci Rep. 2017 Sep 6;7:10659. doi: 10.1038/s41598-017-09368-x (PMC5587556; doi:10.1038/s41598-017-09368-x)
Supplement: Supplementary file 1 — Supplementary Figures [file 41598_2017_9368_MOESM1_ESM.pdf]

Increased TCR signal strength in DN thymocytes promotes development of gut TCR$\alpha\beta^{(+)}$CD8$\alpha\alpha^{(+)}$ intraepithelial lymphocytes

Capucine L. Grandjean, Nital Sumaria, Stefania Martin, Daniel J. Pennington*

Blizard Institute, Barts and The London School of Medicine, Queen Mary University of London, London E1 2AT.

*Correspondence should be addressed to DJP: d.pennington@qmul.ac.uk

Supplementary Data S1

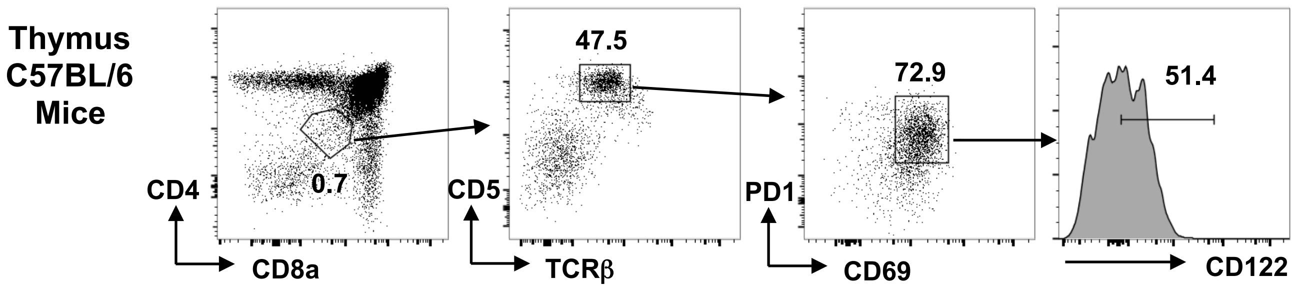

Supplementary Figure S1

Flow cytometry gating strategy used to identify thymic IEL progenitors (as described by *Mayans et al., Immunity 2014, 41 p207-218*)

Supplementary Data S2

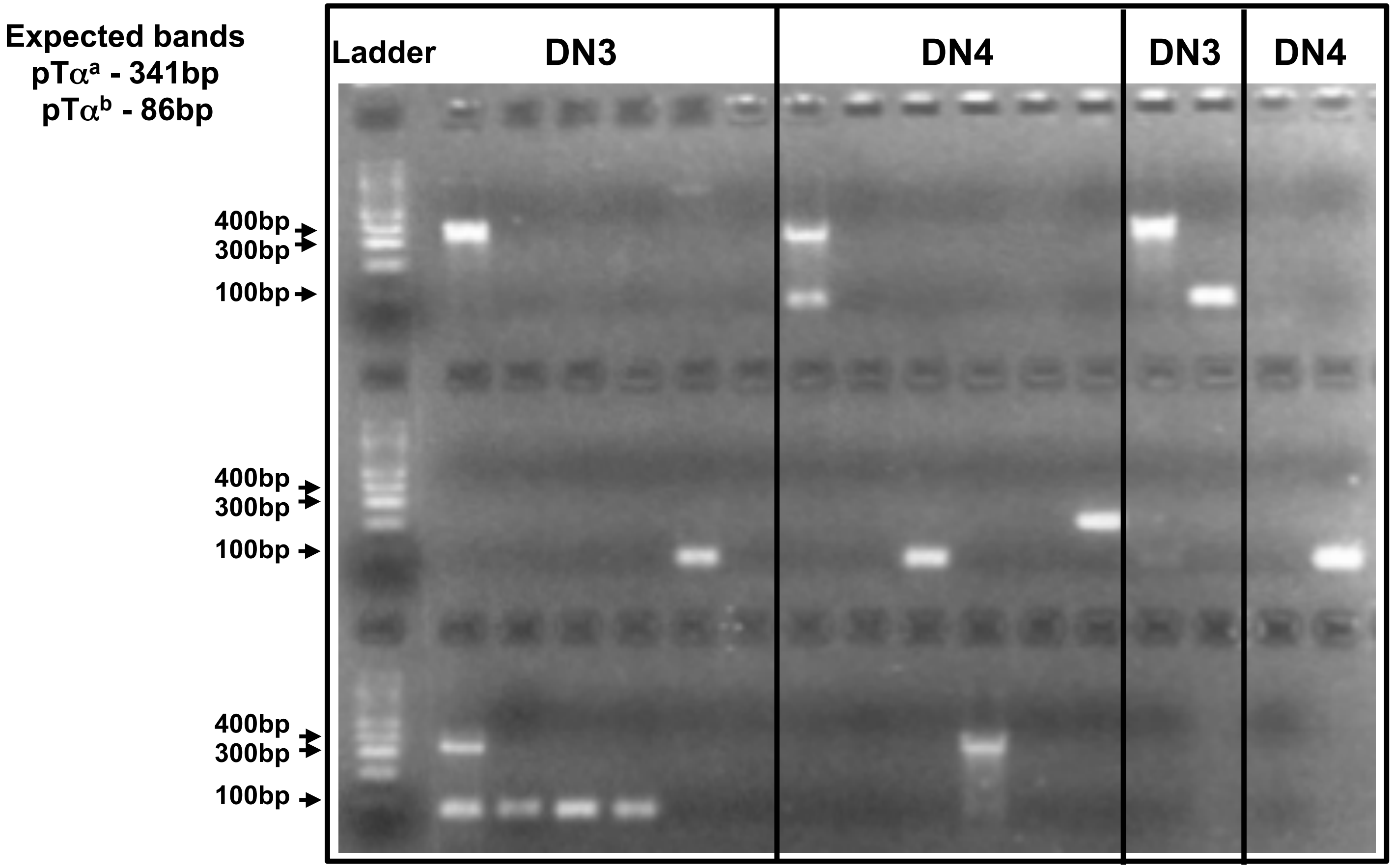

Supplementary Figure S2

Agarose gel from single-cell PCR experiments demonstrating existence of cells expressing pTα[a] only, pTα[b] only, or both isoforms. Protocol and primers are described in methods. Expected bands are 341bp for pTα[a] and 86bp for pTα[b]. Bands were excised and sequenced to confirm identity.

Supplementary Data S3

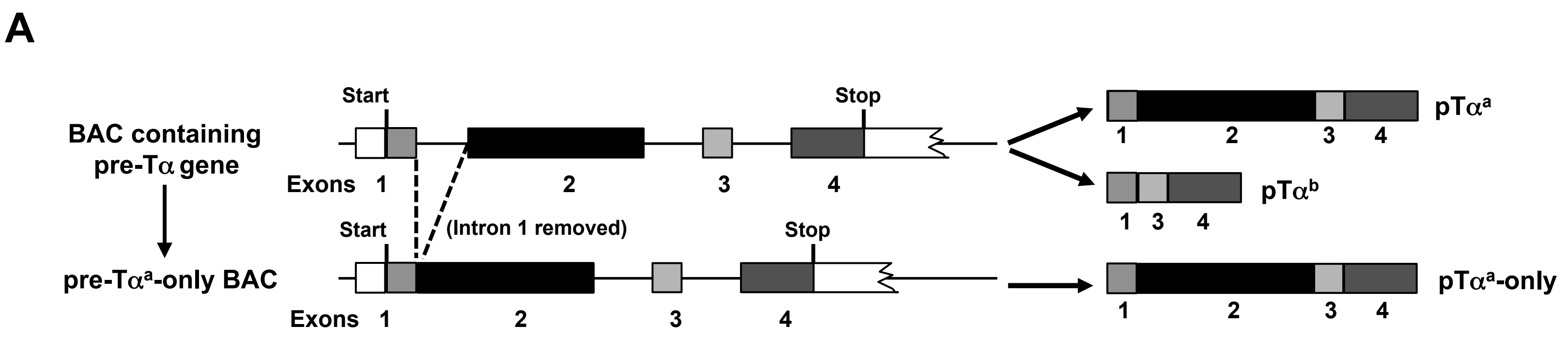

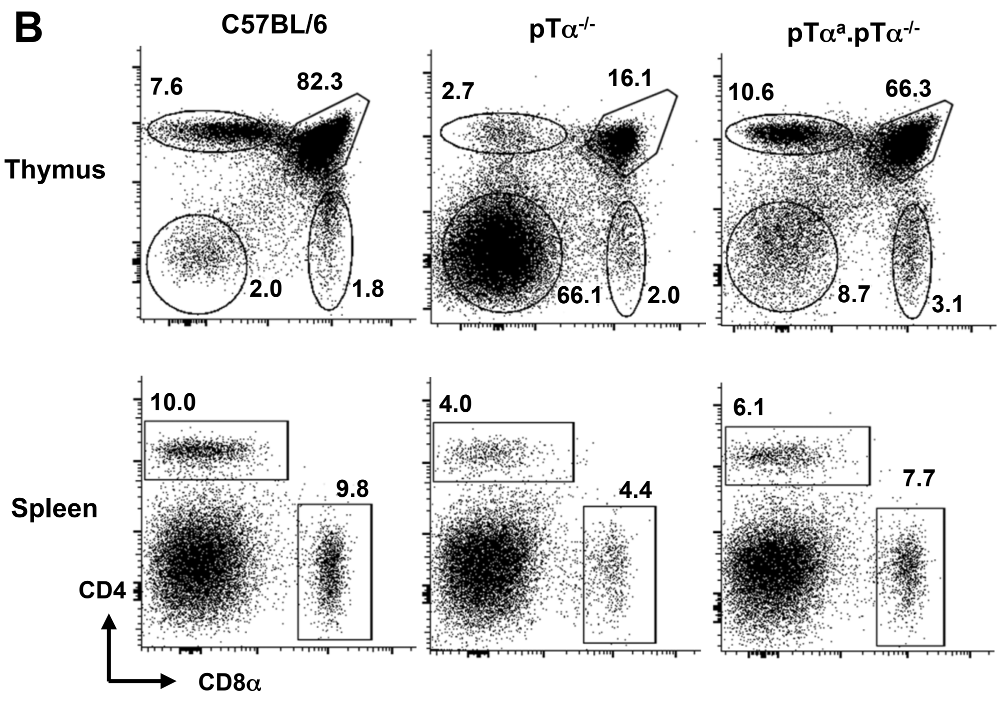

Supplementary Figure S3

(**A**) Schematic representation of the strategy used to generate pTα^a BAC-transgenic mice. (**B**) Representative CD4 versus CD8 flow cytometry plots from thymus and spleen of C57BL/6, pTα-deficient and pTα^a.pT$\alpha^{-/-}$ mice. Percentages of gated cells are indicated.

Supplementary Data S4

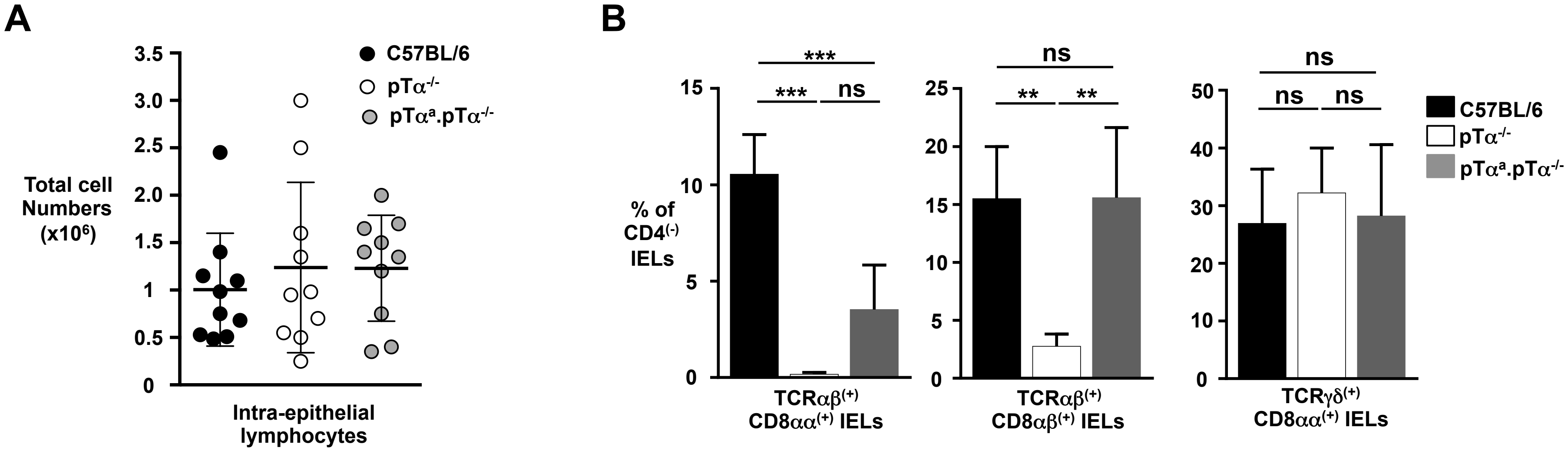

Supplementary Figure S4

(**A**) Total cell yield for IEL-preps from C57BL/6, pTα-deficient and $pT\alpha^{a}.pT\alpha^{-/-}$ mice. (**B**) Summary bar graphs (n>6) of the percentages (of total $CD4^{(-)}$ IELs) of $TCR\alpha\beta^{(+)}CD8\alpha\alpha^{(+)}$, $TCR\alpha\beta^{(+)}CD8\alpha\beta^{(+)}$, and $TCR\gamma\delta^{(+)}CD8\alpha\alpha^{(+)}$ IELs from C57BL/6, pTα-deficient and $pT\alpha^{a}.pT\alpha^{-/-}$ mice. *** $p < 0.001$, **$p < 0.01$, ns is for not significant.

Supplementary Information S5

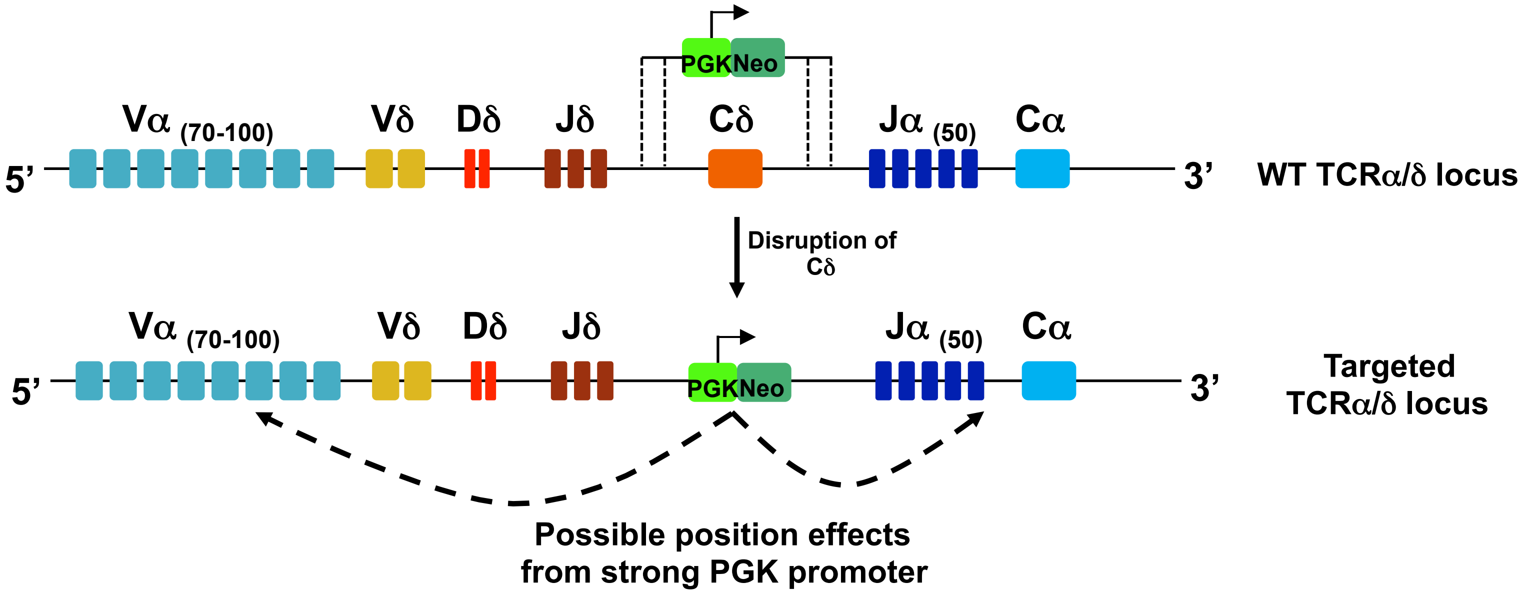

Supplementary Figure S5

Schematic overview of the targeted TCRδ locus in TCRδ$^{-/-}$ mice.
